# Evidence for a Notch1‐mediated transition during olfactory ensheathing cell development

**DOI:** 10.1111/joa.12494

**Published:** 2016-06-06

**Authors:** Sophie R. Miller, Surangi N. Perera, Cristina Benito, Simon R. W. Stott, Clare V. H. Baker

**Affiliations:** ^1^ Department of Physiology, Development and NeuroscienceUniversity of CambridgeCambridgeUK; ^2^ Department of Cell and Developmental BiologyUniversity College LondonLondonUK; ^3^ John van Geest Centre for Brain RepairUniversity of CambridgeCambridgeUK; ^4^ Present address: DanStemUniversity of Copenhagen3B BlegdamsvejDK‐2200Copenhagen NDenmark

**Keywords:** Notch, OEC, olfactory, chick, mouse, human

## Abstract

Olfactory ensheathing cells (OECs) are a unique glial population found in both the peripheral and central nervous system: they ensheath bundles of unmyelinated olfactory axons from their peripheral origin in the olfactory epithelium to their central synaptic targets in the glomerular layer of the olfactory bulb. Like all other peripheral glia (Schwann cells, satellite glia, enteric glia), OECs are derived from the embryonic neural crest. However, in contrast to Schwann cells, whose development has been extensively characterised, relatively little is known about their normal development *in vivo*. In the Schwann cell lineage, the transition from multipotent Schwann cell precursor to immature Schwann cell is promoted by canonical Notch signalling. Here, *in situ* hybridisation and immunohistochemistry data from chicken, mouse and human embryos are presented that suggest a canonical Notch‐mediated transition also occurs during OEC development.

## Introduction

Olfactory ensheathing cells (OECs) ensheath bundles of unmyelinated olfactory axons from their peripheral origin in the olfactory epithelium to their central synaptic targets in the glomerular layer of the olfactory bulb (reviewed by Ekberg et al. 2012). OECs are particularly attractive candidates for transplantation‐mediated repair of the injured central nervous system (CNS): they not only promote axon growth, remyelinate axons, phagocytose debris and stimulate angiogenesis, but also (unlike Schwann cells, the glial cells of all other peripheral nerves) migrate into astrocyte‐rich areas and do not induce the astrocyte stress response (reviewed by Roet & Verhaagen, 2014). They are also being investigated for peripheral nerve repair (reviewed by Radtke & Kocsis, 2014).

Like Schwann cells and satellite glia (reviewed by Woodhoo & Sommer, 2008; Jacob, 2015; Jessen et al. 2015), OECs are derived from the neural crest (Barraud et al. 2010). Hence, one possible future route for generating large numbers of patient‐specific OECs for CNS repair would be to expand and differentiate the neural crest stem cells that persist in skin and hair follicles (Toma et al. 2001; Wong et al. 2006; Hunt et al. 2009, 2010). Schwann cell development has been well characterised (reviewed by Woodhoo & Sommer, 2008; Jacob, 2015; Jessen et al. 2015), and myelinating Schwann cells have been generated in culture from adult skin‐derived rodent and human neural crest stem cells (McKenzie et al. 2006; Sakaue & Sieber‐Blum, 2015). In contrast, relatively little is known about the normal pathway of OEC differentiation, although there are many similarities with Schwann cell development. Characteristic Schwann cell lineage markers such as Ngfr (p75^NTR^, the low‐affinity neurotrophin receptor), the early glial marker Fabp7 (Bfabp/Blbp; brain fatty acid‐binding protein/brain lipid‐binding protein) and Mpz (P0, myelin protein zero) have been identified in developing chicken and rodent OECs by immunostaining (Norgren et al. 1992; Kurtz et al. 1994; Drapkin & Silverman, 1999; Lee et al. 2001; Murdoch & Roskams, 2007; Barraud et al. 2010; Blanchart et al. 2011; Barraud et al. 2013; Pingault et al. 2013). The high‐mobility group (HMG) domain transcription factor Sox10, which is required for the formation of Schwann cells and satellite glia (Britsch et al. 2001; Paratore et al. 2001), is also expressed by developing OECs and required for their normal differentiation (Barraud et al. 2010, 2013; Pingault et al. 2013). During Schwann cell and satellite glial cell development, Sox10 directly activates the *Mpz* promoter (Peirano et al. 2000; Jacob et al. 2014) by recruiting the histone deacetylases Hdac1 and Hdac2 (Jacob et al. 2014). Sox10 also activates the *Fabp7* promoter, but this is likely to be indirect (Jacob et al. 2014; reviewed by Jacob, 2015).

In spinal nerves, Schwann cell development involves the progression of neural crest‐derived cells through two transitional, antigenically distinct embryonic stages. The first is the formation of Fabp7‐positive, Mpz‐positive Schwann cell precursors [seen in mouse hindlimb nerves at embryonic day (E)12–13; in rat hindlimb nerves at E14–15; Jessen et al. 1994; Dong et al. 1999]. Schwann cell precursors require axon‐derived Neuregulin1 type III for their survival (reviewed by Woodhoo & Sommer, 2008; Jessen et al. 2015). They are in fact multipotent progenitor cells, giving rise during normal development not only to Schwann cells, but also to endoneurial fibroblasts, melanocytes, parasympathetic neurons and odontoblasts (Joseph et al. 2004; Adameyko et al. 2009; Dyachuk et al. 2014; Espinosa‐Medina et al. 2014; Kaukua et al. 2014).

In Schwann cell development, the Schwann cell precursor stage is followed by the immature Schwann cell stage, characterised by upregulation of glial fibrillary acidic protein and S100 calcium‐binding protein beta (generated in mouse hindlimb nerves during E13–15; in rat hindlimb nerves during E15–E17; Dong et al. 1999; Jessen & Mirsky, 2005). Unlike Schwann cell precursors, immature Schwann cells can support their own survival via autocrine signalling (reviewed by Woodhoo & Sommer, 2008; Jessen et al. 2015). The formation of mature myelinating and non‐myelinating Schwann cells occurs postnatally in rodents (reviewed by Jessen & Mirsky, 2005; Woodhoo & Sommer, 2008).

The transition from multipotent Schwann cell precursor to immature Schwann cell is promoted by canonical Notch signalling (Woodhoo et al. 2009). In the canonical Notch pathway, the transmembrane ligand on a neighbouring cell interacts with the Notch receptor, triggering two steps of proteolytic cleavage that release the Notch intracellular domain (NICD) from the membrane (reviewed by Andersson et al. 2011; Hori et al. 2013). The NICD translocates into the nucleus where it forms a transcriptional complex with the transcription factor Rbpj and the coactivator Maml1, triggering the expression of target genes including the *Hairy and enhancer of split* (*Hes*) family (reviewed by Kageyama et al. 2007). Conditional Notch inactivation in Schwann cell precursors, using *Desert hedgehog* (*Dhh*) regulatory sequences to drive the Cre‐mediated deletion of *Rbpj* or *Notch1*, delays the formation of immature Schwann cells (Woodhoo et al. 2009). Conversely, conditional Notch activation in Schwann cell precursors, using *Dhh*‐Cre to drive NICD expression, accelerates the formation of immature Schwann cells (Woodhoo et al. 2009).

Here, it was investigated whether Notch signalling might be similarly important during OEC development, by analysing the expression of multiple Notch pathway genes and activated Notch1 during chicken OEC development, and the expression of selected Notch pathway genes in mouse and human embryos. The current results suggest that there is a Notch‐mediated transition during OEC development, from an early Mpz‐positive, ‘OEC precursor’ stage to a more mature stage that in chicken embryos expresses the Notch target gene *Sox2*.

## Materials and methods

### Chicken and mouse embryo collection and sectioning

Fertilised chicken (*Gallus gallus domesticus*) eggs were obtained from commercial sources. Embryos were fixed in modified Carnoy's (six volumes ethanol, three volumes 37% formaldehyde, one volume glacial acetic acid), dehydrated into ethanol, cleared in Histosol (National Diagnostics) and embedded in paraffin wax for sectioning at 6 μm on a rotary microtome (Microm). Wild‐type mouse embryos were collected and immersion‐fixed overnight in 4% paraformaldehyde in phosphate‐buffered saline (PBS) at 4 °C. Embryos were cryoprotected in sucrose, embedded in O.C.T. compound (Tissue Tek) and flash frozen in isopentane on dry ice. Ten‐micrometre sections were taken on a rotary cryostat (Bright Instrument Company).

### Human foetal tissue collection, sectioning and cDNA synthesis

Ethical approval for the use of post mortem human foetal nasal tissue was given by the National Research Ethics Service Committee East of England – Cambridge Central, UK. The tissue was collected at Addenbrooke's Hospital (Cambridge, UK) from patients who had requested pregnancy terminations, and dissected at the John van Geest Centre for Brain Repair, University of Cambridge, Cambridge, UK. Tissue comprising the cartilage of the nasal septum, the medial zygomatic region, the maxillae of the developing upper jaw and the base of the orbital cavities was dissected in HIBERNATE media (Gibco) from the facial region of 7–8‐week embryos.

For cryosectioning, the tissue was immersion‐fixed overnight in 4% paraformaldehyde in PBS at 4 °C, then cryoprotected in 30% diethylpyrocarbonate‐treated sucrose, embedded in O.C.T. compound (Tissue Tek), flash frozen in isopentane on dry ice and stored at −80 °C. Ten‐micrometre sections were taken on a rotary cryostat (Bright Instrument Company).

For cDNA synthesis, the tissue was transferred to the lysis buffer from the Arcturus PicoPure RNA extraction kit (ThermoFisher Scientific), and then to Trizol (Invitrogen) for homogenisation and total RNA extraction according to the manufacturer's instructions. Single‐strand cDNA was generated using Invitrogen's Superscript III First‐Strand Synthesis System kit.

### *In situ* hybridisation

Chicken *Hes4* (*Hairy1*), *Jag1*,* Jag2*,* Notch1* and *Lfng* were kind gifts of Nicolas Daudet (University College London, UK). Fragments of mouse *Jag1*,* Jag2* and *Notch1* and of human *Jag1* and *Notch1* were cloned by polymerase chain reaction (PCR; Table 1) from, respectively, E12.5 mouse cDNA (kind gift of Perrine Barraud, Dept. Physiology, Development and Neuroscience, University of Cambridge, UK) and cDNA prepared from 7‐week‐old dissected human foetal facial region (see previous section). Primer‐BLAST software from NCBI (Ye et al. 2012) was used to design appropriate PCR primers (Table 1) and check them for specificity. The oligonucleotide properties calculator program OligoCalc (Kibbe, 2007; http://www.basic.northwestern.edu/biotools/oligocalc.html) was used to check the melting temperature and self‐complementarity of the primers. cDNA fragments were amplified by PCR and the products cloned into pDrive (Qiagen) using the Qiagen PCR cloning kit. Sequencing was performed by the Biochemistry Department DNA Sequencing Facility, University of Cambridge (Cambridge, UK). Digoxigenin‐labelled antisense riboprobes were generated using standard methods (Henrique et al. 1995) and *in situ* hybridisation performed on cryosections as previously described (O'Neill et al. 2007), except that the slides were not treated with proteinase K.

**Table 1:** PCR cloning information for mouse and human Notch pathway cDNA fragments

Gene	NCBI reference sequence	Forward primer	Reverse primer	Base‐pairs covered
Mouse *Jag1*	NM_013822.5	CCAGTGTCAGAATGACGCCT	TCGACATGTACCCCCGTAGT	1631–2312
Mouse *Jag2*	NM_010588.2	AACGGTGGCACATGCTATGA	ATTCCAGAGCAGATAGCGCC	2178–3001
Mouse *Notch1*	NM_008714.3	TTGACGTCACTCTCCTGTGC	AAGTGCGGGCATCATTCTCA	3593–4318
Human *Jag1*	NM_000214.2	TCACGGGAAGTGCAAGAGTC	CCCATGGTGATGCAAGGTCT	2319–3116
Human *Notch1*	NM_017617.3	GGGTACAAGTGCGACTGTGA	TGACATCGTGCTGGCAGTAG	2206–3067

### Immunohistochemistry

Immunohistochemistry was performed as previously described (Lassiter et al. 2007), except that primary and secondary antibody solutions contained 10% heat‐inactivated sheep or donkey serum for blocking. Primary antibodies used were: anti‐Elavl3/4 (HuC/D; mouse IgG2b, Invitrogen; 1 : 500); anti‐FABP7 (BLBP; rabbit, Merck Millipore ABN14; 1 : 1000); anti‐activated Notch1 (rabbit, Abcam ab8925; 1 : 150); anti‐SOX2 (goat, Santa Cruz Biotechnology, sc‐17320, 1 : 500; rabbit, Abcam ab92494; 1 : 300); anti‐Sox10 (rabbit, gift from Vivian Lee, Medical College of Wisconsin, USA; 1 : 1000); anti‐Tubb3 (clone TUJ1, mouse IgG2a, Covance; 1 : 500). Appropriately matched Alexa Fluor 488‐, 568‐ or 594‐conjugated secondary antibodies, Alexa Fluor 350‐NeutrAvidin and Alexa Fluor 488‐streptavidin were obtained from Molecular Probes/Invitrogen, and biotinylated secondary antibodies from Southern Biotech.

## Results

### *Notch1* is upregulated in developing chicken OECs from E5.5

**Figure 1:**
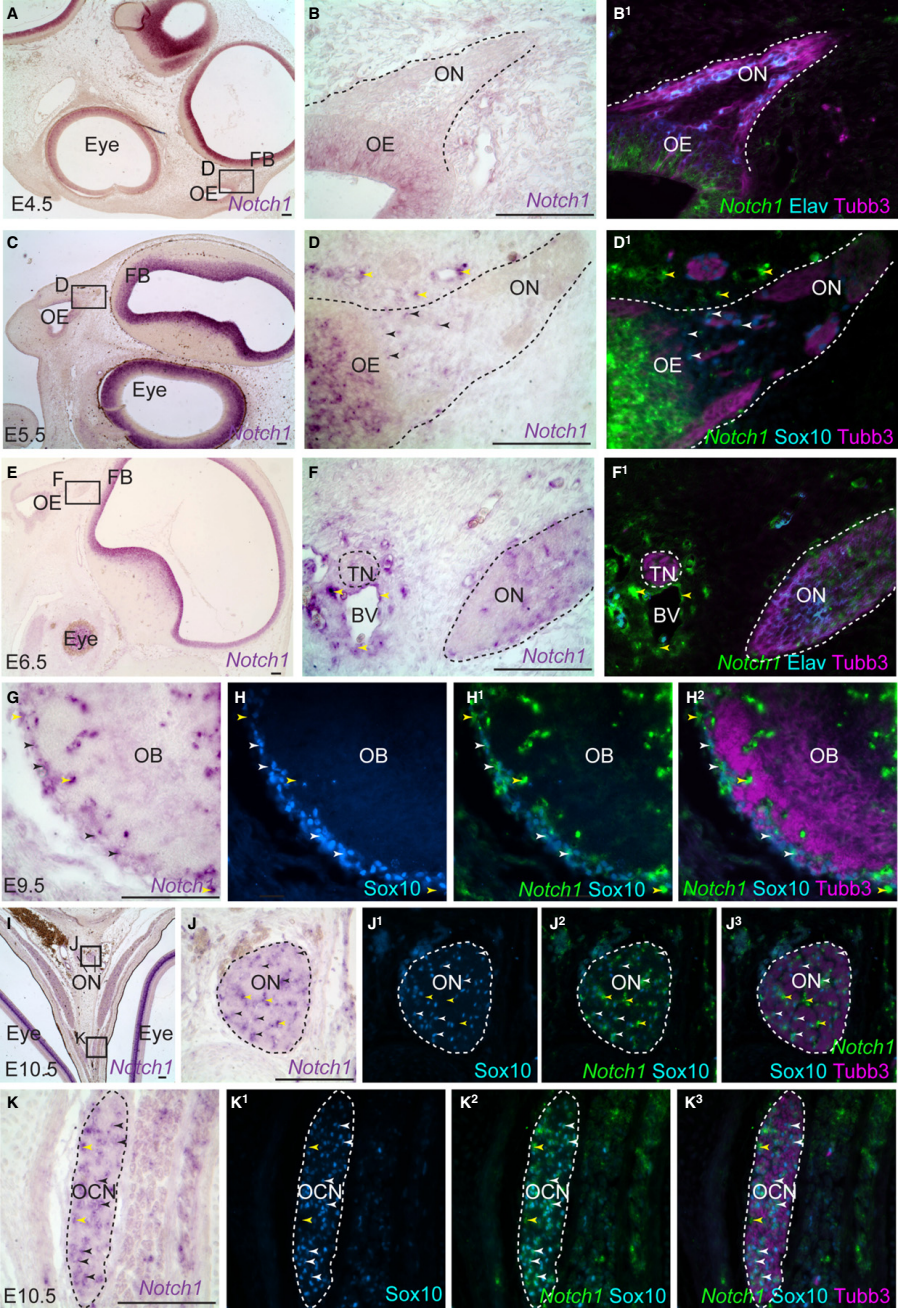
*Notch1* is upregulated in developing chicken OECs from E5.5. Parasagittal (A–D^1^) and coronal (E–K^3^) sections of the embryonic chicken olfactory system. (A) At E4.5, *Notch1* expression is seen in the eye and brain. (B) Higher‐power view of boxed region in (A), showing *Notch1* expression in the apical olfactory epithelium, but no convincing above‐background expression on the olfactory nerve. (B^1^) Same section as (B) immunostained for Tubb3‐positive axons and Elavl3/4‐positive neuronal cell bodies, with a false‐colour overlay of *Notch1*. (C–D^1^) At E5.5, *Notch1* is expressed in a few Sox10‐positive developing OECs (black/white arrowheads) and also around developing blood vessels (yellow arrowheads). (E–F^1^) At E6.5, *Notch1* is expressed by non‐neuronal (Elavl3/4‐negative) cells on the olfactory nerve, presumably developing OECs, and by Schwann cell precursors on the adjacent trigeminal nerve. *Notch1* is also expressed by cells in the walls of nearby blood vessels (yellow arrowheads). (G–G^3^) At E9.5, *Notch1* is expressed by Sox10‐positive OECs in the olfactory nerve layer surrounding the olfactory bulb (black/white arrowheads), and also in cells associated with small blood vessels within the olfactory nerve layer (yellow arrowheads). (H–I^3^) At E10.5, immunostaining for Sox10 confirms that *Notch1* is expressed by OECs on the olfactory nerve (black/white arrowheads highlight examples). *Notch1* is also expressed by Sox10‐negative cells located in the larger gaps between the Tubb3‐positive axon bundles (yellow arrowheads), potentially in the walls of the microvasculature of the olfactory nerve. (J–J^3^) Also at E10.5 (on the same section as H–I^3^), *Notch1* expression is seen in many Sox10‐positive Schwann cells on the oculomotor nerve (black/white arrowheads highlight examples), and also in Sox10‐negative cells in gaps between axon bundles (yellow arrowheads), presumably in the walls of the endoneurial vasculature. BV, blood vessel; Elav, Elavl3/4; FB, forebrain; OB, olfactory bulb; OCN, oculomotor nerve; OE, olfactory epithelium; ON, olfactory nerve. Scale bar: 100 μm.

The earliest stage at which developing chicken OECs have been detected is E3.5, when myelin protein zero (Mpz, P0) expression is first seen on the olfactory nerve, about 12 h after the first olfactory axons emerge from the olfactory epithelium at E3 (Drapkin & Silverman, 1999). At E4.5, *in situ* hybridisation on sections revealed faint *Notch1* expression in the apical olfactory epithelium, but no detectable above‐background expression in cells on the olfactory nerve (Fig. 1A–B^1^). These cells include Elavl3/4 (HuC/D)‐positive gonadotropin‐releasing hormone neurons migrating away from the olfactory epithelium (reviewed by Wray, 2010; also see Sabado et al. 2012), as well as developing OECs.

By E5.5, *Notch1* expression is detected in a few Sox10‐positive developing OECs associated with olfactory axons (black/white arrowheads), as well as being strong in the olfactory epithelium and around blood vessels (yellow arrowheads) (Fig. 1C–D^1^). At E6.5, 3 days after Mpz‐positive developing OECs are first detected on the olfactory nerve (Drapkin & Silverman, 1999), *Notch1* is expressed by many non‐neuronal (Elavl3/4‐negative) cells associated with olfactory axons, presumably OECs (Fig. 1E–F^1^). *Notch1* is also expressed by presumptive Schwann cell precursors on adjacent trigeminal nerve branches, and by cells associated with the walls of the developing vasculature (yellow arrowheads, Fig. 1E–F^1^). At E9.5, *Notch1* is expressed by Sox10‐positive OECs in the olfactory nerve layer surrounding the olfactory bulb (black/white arrowheads), and also by cells associated with microvasculature within the olfactory nerve layer (yellow arrowheads) (Fig. 1G–G^3^). At E10.5, *Notch1* is still expressed strongly on the olfactory nerve by Sox10‐positive OECs (black/white arrowheads) and by Sox10‐negative cells in larger gaps between olfactory axon bundles, most likely associated with endoneurial vasculature (yellow arrowheads) (Fig. 1H–I^3^). At the same stage, *Notch1* is expressed by many Sox10‐positive Schwann cells on the oculomotor nerve (black/white arrowheads) and also by Sox10‐negative cells in gaps between axon bundles, presumably around the endoneurial vasculature (yellow arrowheads) (Fig. 1J–J^3^).

The upregulation of *Notch1* expression in the OEC lineage from E5.5, 2 days after Mpz‐positive glial cells are first detected on the olfactory nerve at E3.5 (Drapkin & Silverman, 1999), is reminiscent of the increased expression of *Notch1* mRNA detected by reverse transcriptase‐PCR on embryonic rat hindlimb nerves between E14 (Schwann cell precursor stage) and E18 (immature Schwann cell stage) (Woodhoo et al. 2009). This suggests the possibility that Notch signalling may drive a comparable step in OEC maturation from an early ‘OEC precursor’ stage characterised by expression of Mpz (Norgren et al. 1992; Drapkin & Silverman, 1999).

### Notch1 signalling is active in developing chicken OECs

**Figure 2:**
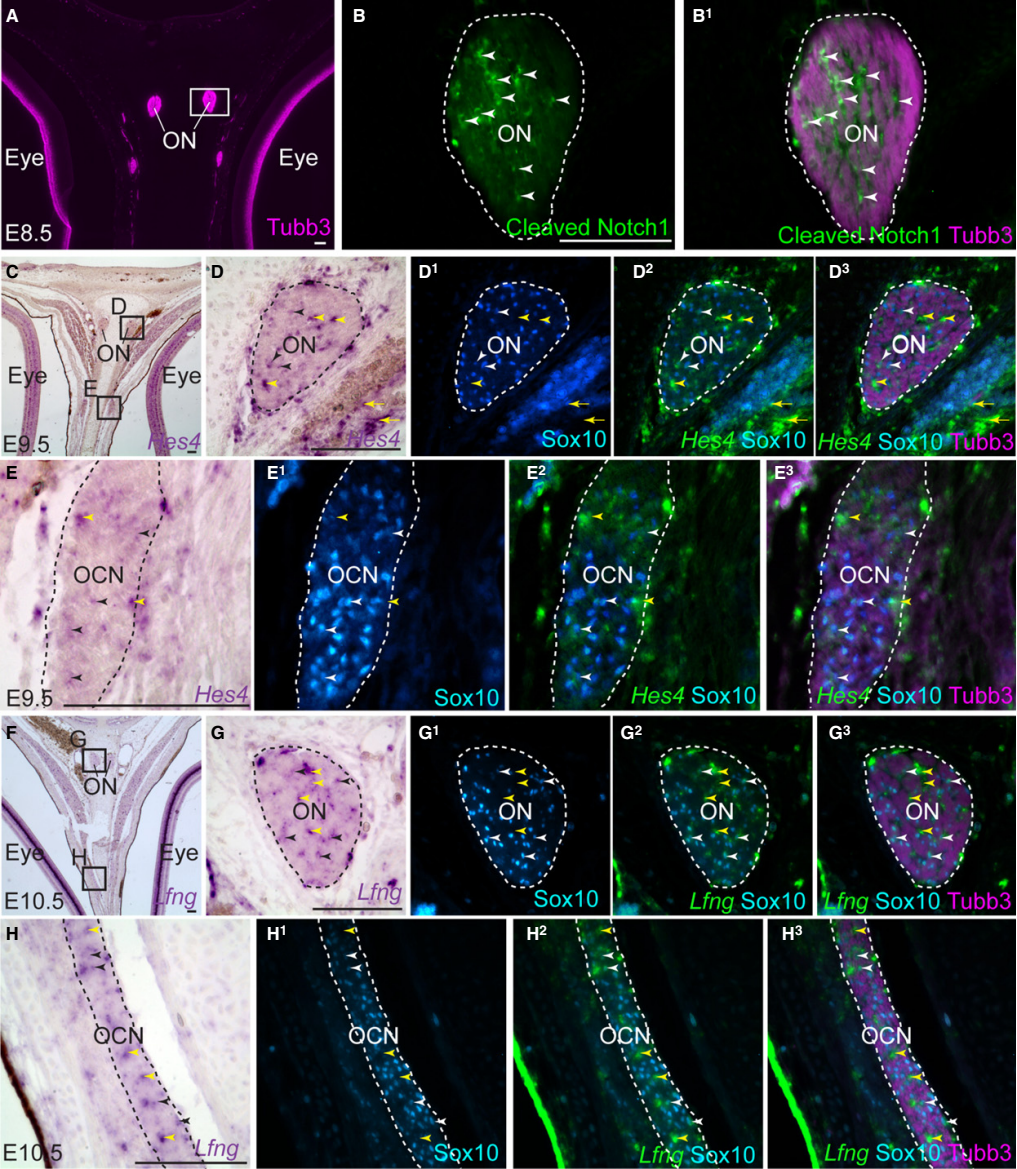
Notch signalling is active in developing chicken OECs. Coronal sections of the embryonic chicken olfactory system. (A–B^1^) At E8.5, immunostaining for Tubb3 and activated (cleaved) Notch1 reveals the presence of activated Notch1 in many cells between the Tubb3‐positive axon bundles of the olfactory nerve, presumably developing OECs. (C–D^3^) At E9.5, the Notch target gene *Hes4* (*Hairy1*) is expressed by a subset of Sox10‐positive OECs on the olfactory nerve (black/white arrowheads). *Hes4* is also expressed by Sox10‐negative cells in the larger gaps between the Tubb3‐positive axon bundles (yellow arrowheads); these may be associated with endoneurial microvasculature, given that *Hes4* is also expressed by cells in the walls of blood vessels (yellow arrowheads highlight examples). (E–E^3^) Also at E9.5 (on the same section as C–D^3^), *Hes4* is expressed by a subset of Sox10‐positive Schwann cells on the oculomotor nerve (black/white arrowheads), in addition to Sox10‐negative cells (yellow arrowheads). (F–G^3^) At E10.5, the Notch target gene *Lfng* is expressed by a subset of Sox10‐positive OECs on the olfactory nerve (black/white arrowheads). *Lfng* is also expressed by Sox10‐negative cells in the larger gaps between the Tubb3‐positive axon bundles (yellow arrowheads), potentially associated with blood vessels. (H–H^3^) On the same section, *Lfng* is expressed by a subpopulation of Sox10‐positive Schwann cells on the oculomotor nerve (black/white arrowheads), and by Sox10‐negative cells, potentially associated with endoneurial microvasculature (yellow arrowheads). OCN, oculomotor nerve; ON, olfactory nerve. Scale bar: 100 μm.

In the rat, increased NICD expression is detected by Western blot between E14 (when Schwann cell precursors are present) and E18 (when immature Schwann cells are present) (Woodhoo et al. 2009). Immunostaining sections with an antibody against activated Notch1, which detects the *N*‐terminal sequence of the cleaved human NICD, suggested that the Notch pathway is active in at least some OECs (located between Tubb3‐positive axon bundles) on the chicken olfactory nerve at E8.5 (Fig. 2A–B^1^).

Further support for active Notch signalling in a subset of chicken OECs is provided by Notch target gene expression in cells on the olfactory nerve and in the olfactory nerve layer of the olfactory bulb. Chicken *Hes4* is a homologue of *Drosophila hairy*, originally named c*Hairy1* (Palmeirim et al. 1997). In humans, HES4 (which is missing in rodents) is the most similar of the seven HES family proteins to HES1, which is encoded by a direct Notch target gene involved in maintaining certain stem and progenitor cells (Kobayashi & Kageyama, 2014). At E9.5, chicken *Hes4* is expressed by a subset of developing OECs and Schwann cells (black/white arrowheads, Fig. 2C–E^3^). This could be the result of oscillatory expression, as seen for Hes1 (Hirata et al. 2002). *Hes4* is also expressed by Sox10‐negative cells on both the olfactory nerve and other peripheral nerves (yellow arrowheads, Fig. 2C–E^3^): these may be associated with the endoneurial vasculature, given that *Hes4* is also expressed by cells in the walls of nearby blood vessels (yellow arrows, Fig. 2D–D^3^).

*Lunatic fringe* (*Lfng*) is another Notch target gene, encoding a glycosyltransferase that regulates Notch pathway activity via glycosylation of the Notch receptor in the Golgi apparatus (reviewed by Takeuchi & Haltiwanger, 2014). *In situ* hybridisation for *Lfng* on the olfactory nerve at E10.5 (Fig. 2F–G^3^) reveals a more sparse expression pattern than *Notch1* (compare with Fig. 1I–I^3^, which shows a nearby section from the same embryo). Indeed, *Lfng* is expressed by only a subset of Sox10‐positive OECs (black/white arrowheads, Fig. 2G–G^3^). *Lfng* is also expressed by Sox10‐negative cells in larger gaps between Tubb3‐positive olfactory axon bundles, presumably around developing blood vessels (yellow arrowheads, Fig. 2G–G^3^). *Lfng* transcripts can also be detected at E10.5 in Sox10‐positive Schwann cells on the oculomotor nerve (black/white arrowheads, Fig. 2H–H^3^), and in Sox10‐negative cells in gaps between axon bundles, presumably around the endoneurial vasculature (yellow arrowheads, Fig. 2H–H^3^).

Overall, these results suggest that the Notch signalling pathway is active during chicken OEC development, most likely from the first upregulation of *Notch1* in developing OECs from E5.5, with activity maintained in a subset of OECs until at least E10.5.

### Notch1 signalling in developing chicken OECs may upregulate *Sox2*

Transfection of an activated form of chicken Notch1 into cultured chicken neural crest cells promotes expression of the HMG domain transcription factor Sox2 (Wakamatsu et al. 2004; indeed *Sox2* is a direct target of Notch/Rbpj signalling in mouse neural stem cells; Ehm et al. 2010; Li et al. 2012). Sox2 is expressed by Schwann cell precursors and immature Schwann cells in both chicken and mouse, but its downregulation is necessary for Schwann cell maturation and myelination (Wakamatsu et al. 2004; Le et al. 2005; Adameyko et al. 2012), although weak Sox2 expression is maintained in non‐myelinating Schwann cells (Koike et al. 2014). These results prompted us to investigate Sox2 expression during OEC development.

**Figure 3:**
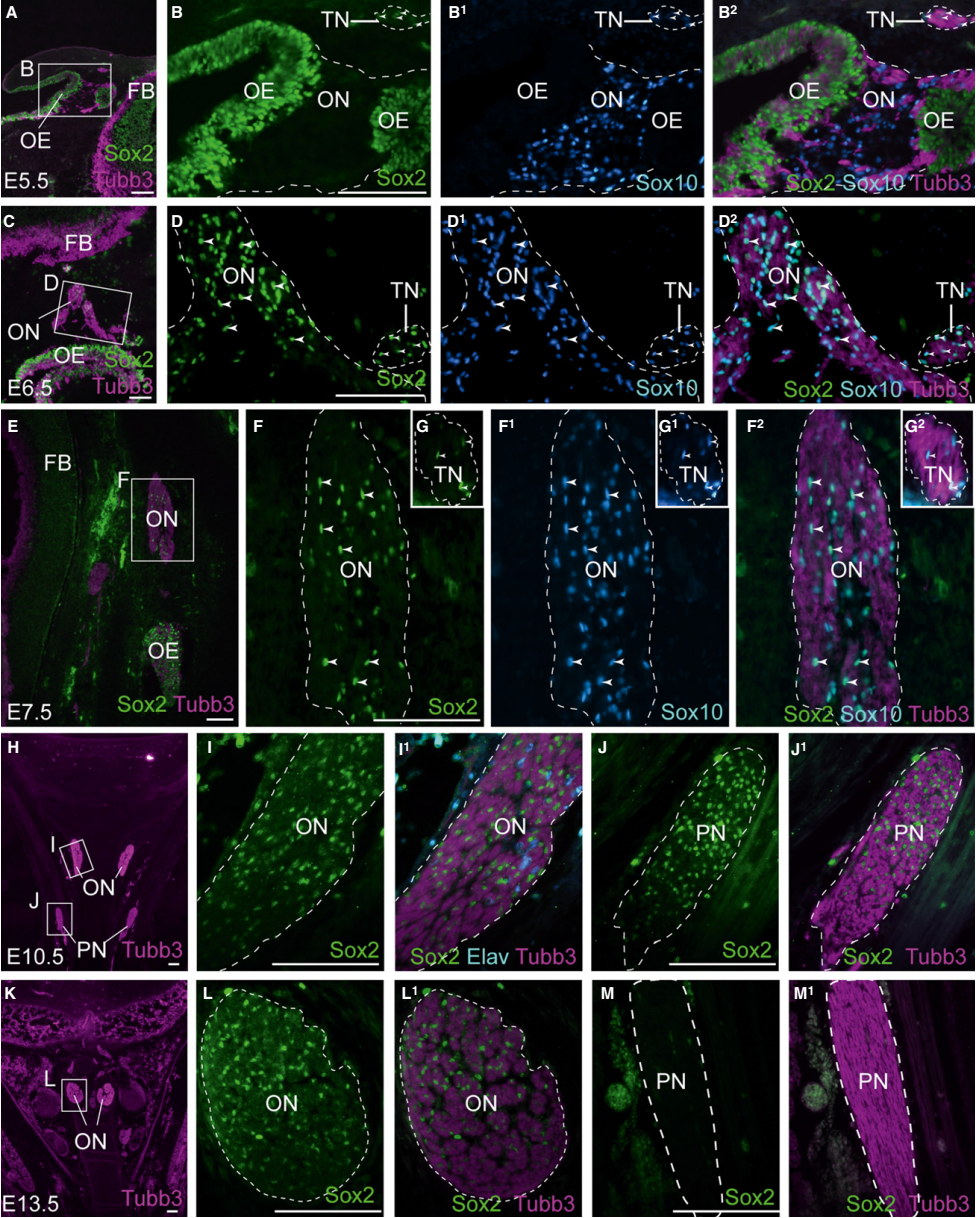
The Notch target Sox2 is upregulated in chicken OECs from E6.5 and maintained until at least E13.5. Parasagittal (A–G^2^) and coronal (H–M^1^) sections of the embryonic chicken olfactory system. (A–B^2^) At E5.5, immunostaining for Sox2, Tubb3‐positive axons and Sox10‐positive OECs reveals absence of Sox2 on the olfactory nerve (B,B^1^), although in a different region of the same section, weak Sox2 expression is detected in Schwann cell precursors on the trigeminal nerve (arrowheads, B–B^2^). (C–D^2^) At E6.5, Sox2 is detected in almost all Sox10‐positive developing OECs on the olfactory nerve, as well as in Sox10‐positive Schwann cells on the trigeminal nerve (arrowheads, D–D^2^). (E–G^2^) At E7.5, Sox2 expression is maintained in Sox10‐positive developing OECs (arrowheads, F–F^2^). Insets in (F–F^2^) show Sox2 expression in Sox10‐positive Schwann cells on the trigeminal nerve from an adjacent tissue section (arrowheads, G–G^2^). (H–J^1^) At E10.5, Sox2 is expressed by non‐neuronal cells on the olfactory nerve, presumably OECs (I,I^1^), and in Schwann cells on a nearby peripheral nerve in the same section (J,J^1^). (K–M^1^) At E13.5, Sox2 expression is maintained in non‐neuronal cells between the bundles of olfactory axons, presumably OECs (L,L^1^), but Sox2 is no longer expressed by Schwann cells on a nearby peripheral nerve in the same section (M,M^1^). Elav, Elavl3/4; FB, forebrain; OB, olfactory bulb; OCN, oculomotor nerve; OE, olfactory epithelium; ON, olfactory nerve; ONL, olfactory nerve layer; PN, peripheral nerve; TN, trigeminal nerve. Scale bar: 100 μm.

At E5.5 (when only a few developing OECs express *Notch1*; Fig. 1C–D^1^), immunostaining revealed no Sox2 expression on the olfactory nerve (Fig. 3A–B^2^), although faint expression was seen in Schwann cell precursors on the trigeminal nerve (arrowheads, Fig. 3B–B^2^). A day later, at E6.5 (Fig. 3C–D^2^), Sox2 is expressed by almost all Sox10‐positive developing OECs on the olfactory nerve, as well as the Sox10‐positive Schwann cells on the adjacent trigeminal nerve (arrowheads, Fig. 3D–D^2^). At E7.5 (Fig. 3E–G^2^), Sox2 expression is maintained in Sox10‐positive developing OECs (arrowheads, Fig. 3F–F^2^), as well as in developing Schwann cells on an adjacent tissue section (arrowheads, Fig. 3G–G^2^). At E10.5 (Fig. 3H–J^1^), Sox2 is expressed by non‐neuronal cells on the olfactory nerve, presumably OECs (Fig. 3I,I^1^), and also in Schwann cells (Fig. 3J,J^1^). At E13.5 (Fig. 3K–M^1^; this is several days after the onset of synapse formation between olfactory receptor neurons and their targets in the olfactory bulb, which occurs at E8–10; Ayer‐Le Lièvre et al. 1995), Sox2 is still expressed by OECs on the olfactory nerve (Fig. 3L,L^1^), but seems to have been downregulated in the Schwann cell lineage (Fig. 3M,M^1^; presumably during the transition from immature to mature Schwann cells; Le et al. 2005).

Overall, these results suggest that in chicken embryos, Notch1 signalling may mediate a transition from a Sox2‐negative OEC precursor stage to a more mature Sox2‐positive OEC stage.

### Chicken olfactory receptor neurons express the Notch ligand genes *Jag1* and *Jag2*

**Figure 4:**
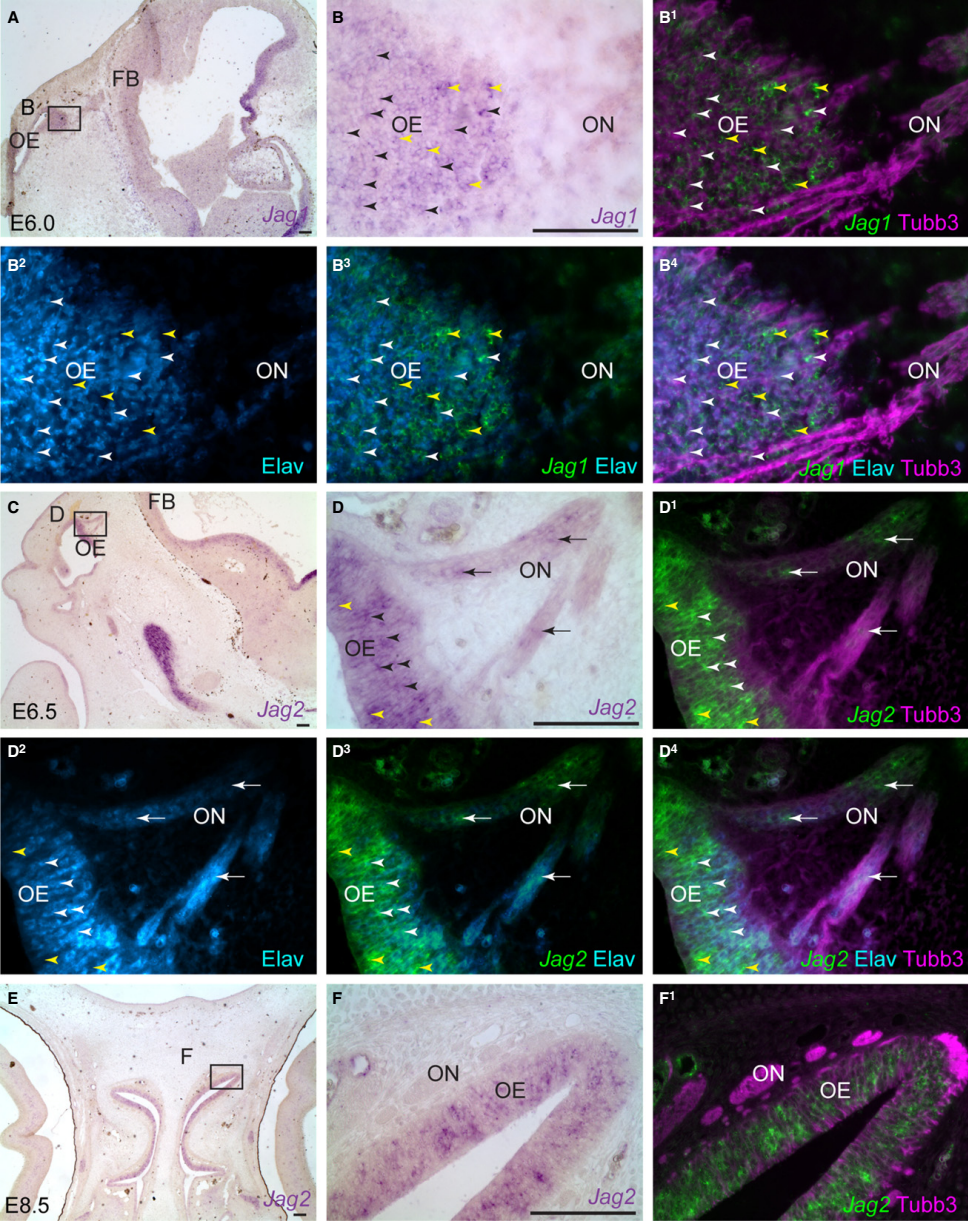
The canonical Notch ligand genes *Jag1* and *Jag2* are expressed by chicken olfactory receptor neurons. Parasagittal sections of the embryonic chicken olfactory system. (A,B) At E6.0, *Jag1* is expressed throughout the olfactory epithelium. (B^1^–B^4^) Same section as (B) immunostained for Tubb3‐positive axons and Elavl3/4‐positive neuronal cell bodies, with a false‐colour overlay of *Jag1*, reveals that *Jag1* is expressed by Elavl3/4‐positive olfactory receptor neurons (black/white arrowheads) and also non‐neuronal supporting cells (yellow arrowheads). (C–D^4^) At E6.5, *Jag2* is expressed by Elavl3/4‐positive olfactory receptor neurons in the intermediate layer (black/white arrowheads), by a few Elavl3/4‐negative apical support cells (yellow arrowheads), and by migrating Elavl3/4‐positive neurons on the olfactory nerve (black/white arrows). *Jag2* expression can also be seen in the vasculature, most likely in endothelial cells. (E–F^1^) At E8.5, *Jag2* expression is maintained in olfactory receptor neurons in the intermediate layer of the olfactory epithelium. Expression is also seen in the vasculature, most likely endothelial cells. Elav, Elavl3/4; FB, forebrain; OE, olfactory epithelium; ON, olfactory nerve. Scale bar: 100 μm.

In the rat, the canonical Notch ligand Jagged1 (Jag1) is expressed on sciatic nerve axons at E14, suggesting this could be involved in Notch signalling during the transition from Schwann cell precursor to immature Schwann cell (Woodhoo et al. 2009). In the chick at E6.0, *Jag1* is expressed by Elavl3/4‐positive olfactory receptor neurons in the olfactory epithelium (black/white arrowheads), as well as in non‐neuronal support cells (yellow arrowheads) (Fig. 4A–B^4^). At E6.5, *Jag2* expression is similarly expressed in Elavl3/4‐positive olfactory receptor neurons in the olfactory epithelium (black/white arrowheads), as well as in non‐neuronal support cells (yellow arrowheads), but is also detected in migratory Elavl3/4‐positive neurons on the olfactory nerve (white arrows) (Fig. 4C–D^4^). *Jag2* expression is maintained in olfactory receptor neurons in the intermediate layer of the olfactory epithelium (Beites et al. 2005) until at least E8.5 (Fig. 4E–F^1^). Since both *Jag1* and *Jag2* are expressed by embryonic chicken olfactory receptor neurons at the stage when *Notch1* is upregulated in developing OECs, these ligands could be available on olfactory axons to activate Notch1 signalling in adjacent glial cells.

### Embryonic mouse and human OECs express *Notch1*, while olfactory receptor neurons express *Jag1/2*

**Figure 5:**
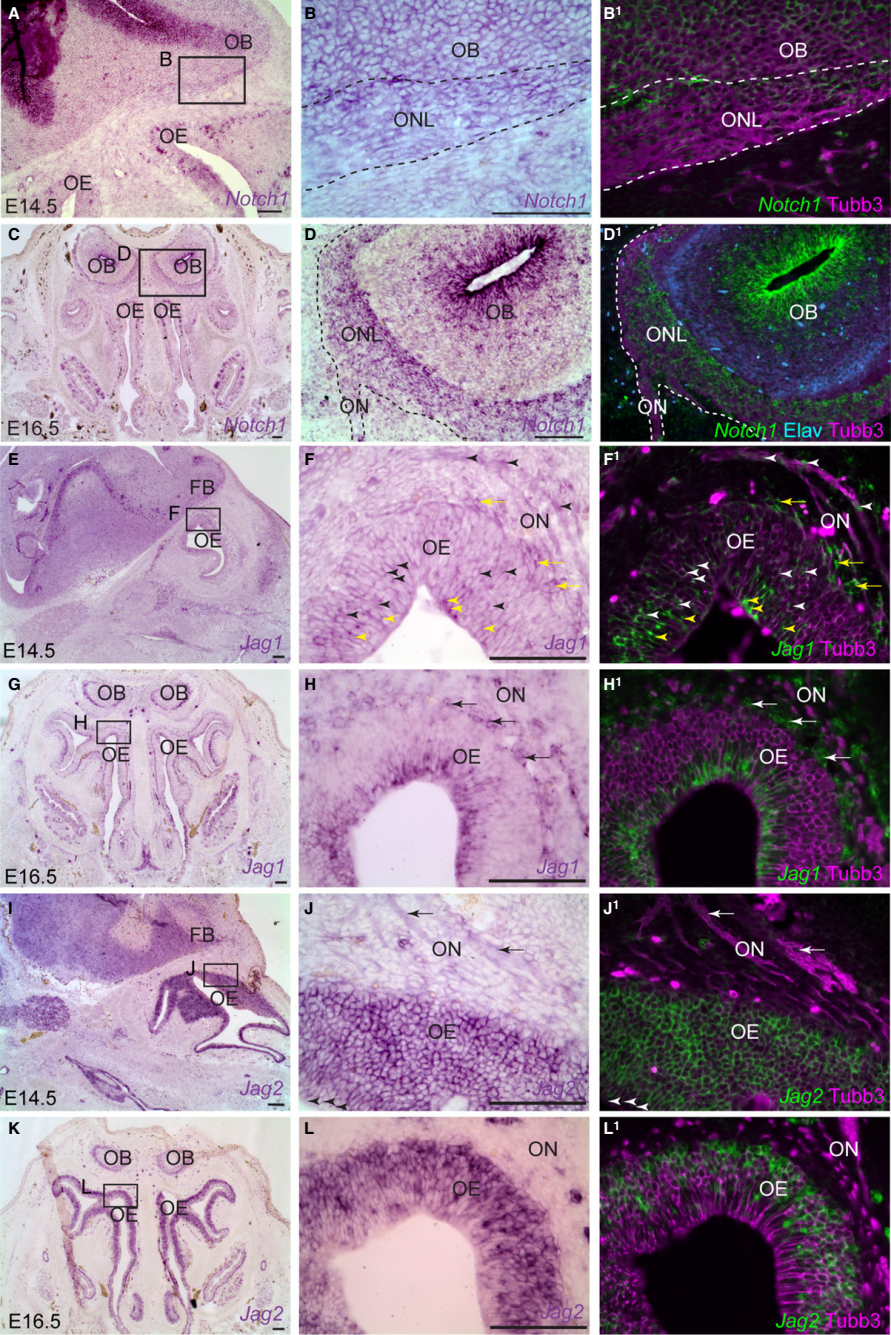
*Notch1* is expressed by embryonic mouse OECs, while *Jag1* and *Jag2* are expressed by olfactory receptor neurons. Sections of the embryonic olfactory system in mouse (E14.5 parasagittal; E16.5 coronal). (A) In E14.5 mouse embryos, *Notch1* is expressed by the basal layer of the olfactory epithelium and in the subventricular zone of the forebrain. (B) Higher‐power view of boxed region in (A), showing expression of *Notch1* in the olfactory nerve layer around the olfactory bulb. (B^1^) Same section as (B) immunostained for Tubb3‐positive axons, with a false‐colour overlay of *Notch1*. (C–D^1^) At E16.5, *Notch1* is expressed by OECs on the olfactory nerve and in the olfactory nerve layer of the olfactory bulb. (E–F^1^) At E14.5, *Jag1* is expressed by Tubb3‐positive olfactory receptor neurons and their axons (black/white arrowheads), in non‐neuronal apical support cells in the olfactory epithelium (yellow arrowheads), and in non‐neuronal cells close to the olfactory epithelium, likely mesenchymal cells of the lamina propria (black/white arrows). (G–H^1^) By E16.5, *Jag1* expression is restricted to apical (sustentacular) cells in the olfactory epithelium and to non‐neuronal cells apposed to the olfactory epithelium and near, but not directly associated with, Tubb3‐positive olfactory axons (most likely mesenchymal cells of the lamina propria, arrows). [NB Expression around the olfactory bulbs, seen at low‐power in (G), is not within the olfactory nerve layer but deep to it.] (I–J^1^) At E14.5, *Jag2* is expressed by all cells in the olfactory epithelium, though more weakly in the most apical cells (arrowheads, J,J^1^). *Jag2* expression can also be detected in olfactory axons (arrows). In contrast to *Jag1*,* Jag2* is not expressed by mesenchymal cells in the lamina propria (compare with F,F^1^). (K–L^1^) By E16.5, expression of *Jag2* is essentially reciprocal to *Jag1*: it remains strong in olfactory receptor neurons in the intermediate layer, and in basal progenitors, but is more weakly expressed by the most apical layer. No expression is seen in mesenchymal cells in the lamina propria (compare with H,H^1^). [NB Expression around the olfactory bulbs, seen at low‐power in (K), is not within the olfactory nerve layer but deep to it.] FB, forebrain; OB, olfactory bulb; OE, olfactory epithelium; ON, olfactory nerve. Scale bar: 100 μm.

In the mouse, *Notch1* is expressed by OECs on the olfactory nerve and in the olfactory nerve layer of the olfactory bulb at both E14.5 and E16.5 (Fig. 5A–D^1^).

A complementary expression pattern for *Jag1* and *Jag2* was previously reported in the mouse olfactory epithelium at E12.5, with *Jag1* mostly confined to apical cells, and *Jag2* expressed weakly in apical cells and more strongly in intermediate and basal progenitors (Cau et al. 2002). A similar complementary expression pattern was found here at both E14.5 and E16.5. At E14.5, *Jag1* is expressed apically in the olfactory epithelium, presumably by sustentacular cells, and by at least some olfactory receptor neurons in the intermediate layer (indeed, *Jag1* mRNA seems to be present within olfactory axons themselves) (Fig. 5E–F^1^). By E16.5, however, *Jag1* expression is restricted to the non‐neuronal apical layer of the olfactory epithelium (Fig. 5G–H^1^), where sustentacular cells are located (Beites et al. 2005). In contrast, *Jag2* is strongly expressed by Tubb3‐positive olfactory receptor neurons in the intermediate layer at both E14.5 and E16.5 (*Jag2* mRNA also seemed to be present in olfactory axons), but is more weakly expressed by the apical layer of sustentacular cells (Fig. 5I–L^1^). Thus, *Jag1* and *Jag2* are expressed by olfactory receptor neurons at E14.5, with *Jag2* (though not *Jag1*) expression maintained until at least E16.5. Hence, these ligands could be available on olfactory axons to activate Notch1 in developing mouse OECs.

**Figure 6:**
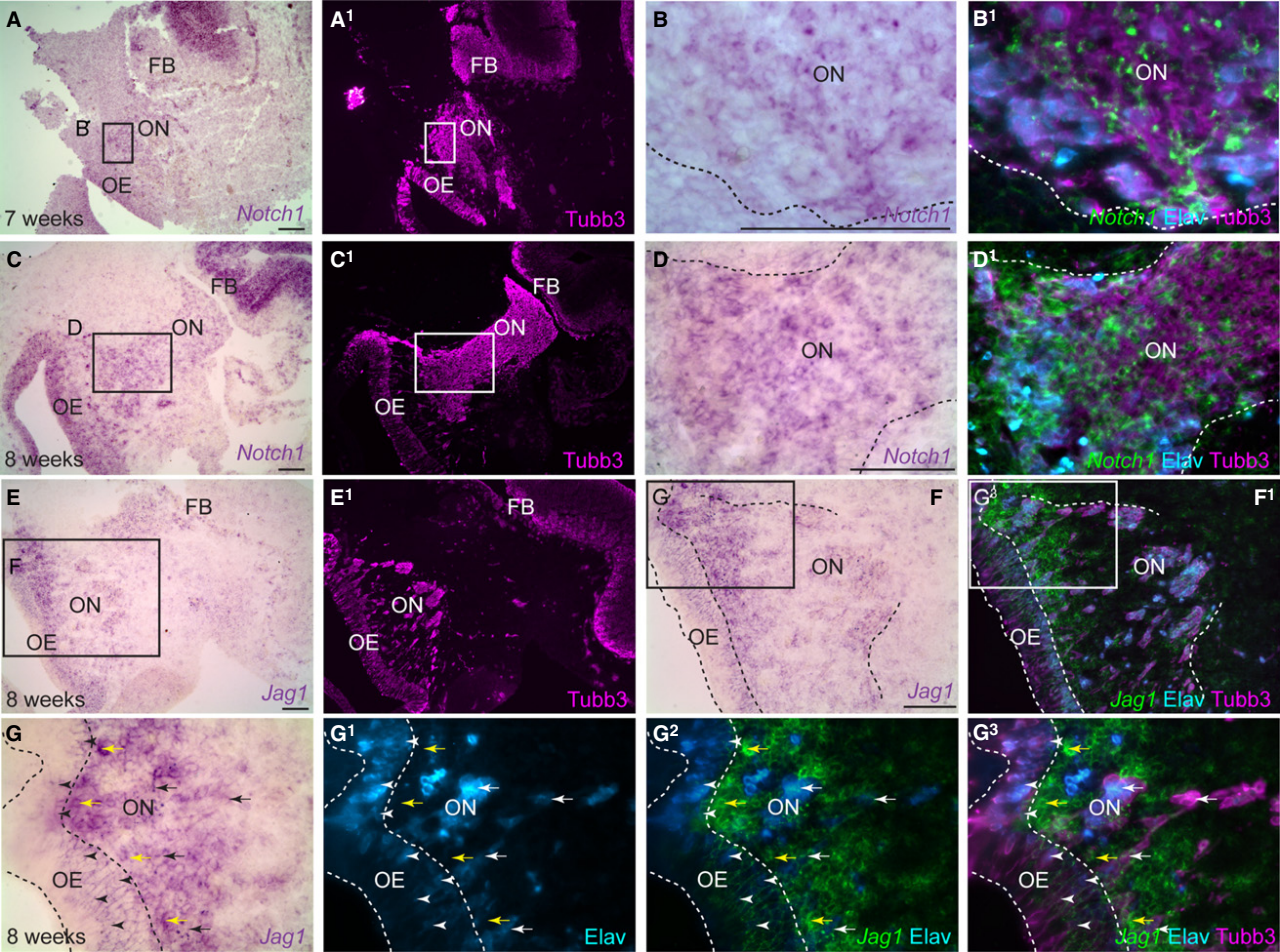
*Notch1* is expressed by embryonic human OECs, while *Jag1* is expressed by olfactory receptor neurons. Parasagittal sections of the human embryonic olfactory system. (A) At 7 weeks of development, *Notch1* is expressed by the subventricular zone of the forebrain. (A^1^) Same section as (A) showing Tubb3 immunostaining for axons. (B) Higher‐power view of boxed region in (A), showing expression of *Notch1* on the olfactory nerve. (B^1^) Same section as (B) immunostained for Tubb3‐positive axons and Elavl3/4‐positive neurons, with a false‐colour overlay of *Notch1*, shows that *Notch1* is expressed by non‐neuronal cells on the olfactory nerve, presumably developing OECs. (C–D^1^) At 8 weeks, *Notch1* is expressed by non‐neuronal cells on the olfactory nerve, presumably developing OECs. (E–G^3^) At 8 weeks of development, *Jag1* can be detected in Elavl3/4‐positive olfactory receptor neurons in the intermediate and basal layers of the olfactory epithelium (black/white arrowheads), which might be newly differentiated olfactory receptor neurons and/or precursors of the *Jag1*‐positive migratory neurons on the olfactory nerve (black/white arrows). *Jag1* expression is also seen in non‐neuronal mesenchymal cells in the lamina propria (yellow arrows). BV, blood vessel; FB, forebrain; OB, olfactory bulb; OE, olfactory epithelium; ON, olfactory nerve. Scale bar: 100 μm.

*Notch1* expression was also identified in cells on the human olfactory nerve at both 7 weeks (about Carnegie stage 20, when the olfactory bulb is forming; Bossy, 1980) and 8 weeks of development (Carnegie stage 23, after the olfactory bulb has formed; Bossy, 1980). The *Notch1*‐positive cells are presumably OECs as *Notch1* expression does not overlap with migratory Elavl3/4‐positive neurons (Fig. 6A–D^1^). At 8 weeks of development, the Notch ligand gene *Jag1* is expressed in the intermediate and basal layers of the olfactory epithelium, suggesting expression in both olfactory receptor neurons (black/white arrowheads) and basal progenitors and/or the precursors of emigrating neurons (black/white arrows), which also seem to express *Jag1* (Fig. 6E–G^3^). Furthermore, *Jag1* is detected in non‐neuronal mesenchymal cells of the lamina propria surrounding the emerging olfactory axons (yellow arrows, Fig. 6E–G^3^), which could be another source of Notch pathway activation in developing OECs.

### Embryonic mouse and human OECs do not express the Notch target Sox2

**Figure 7:**
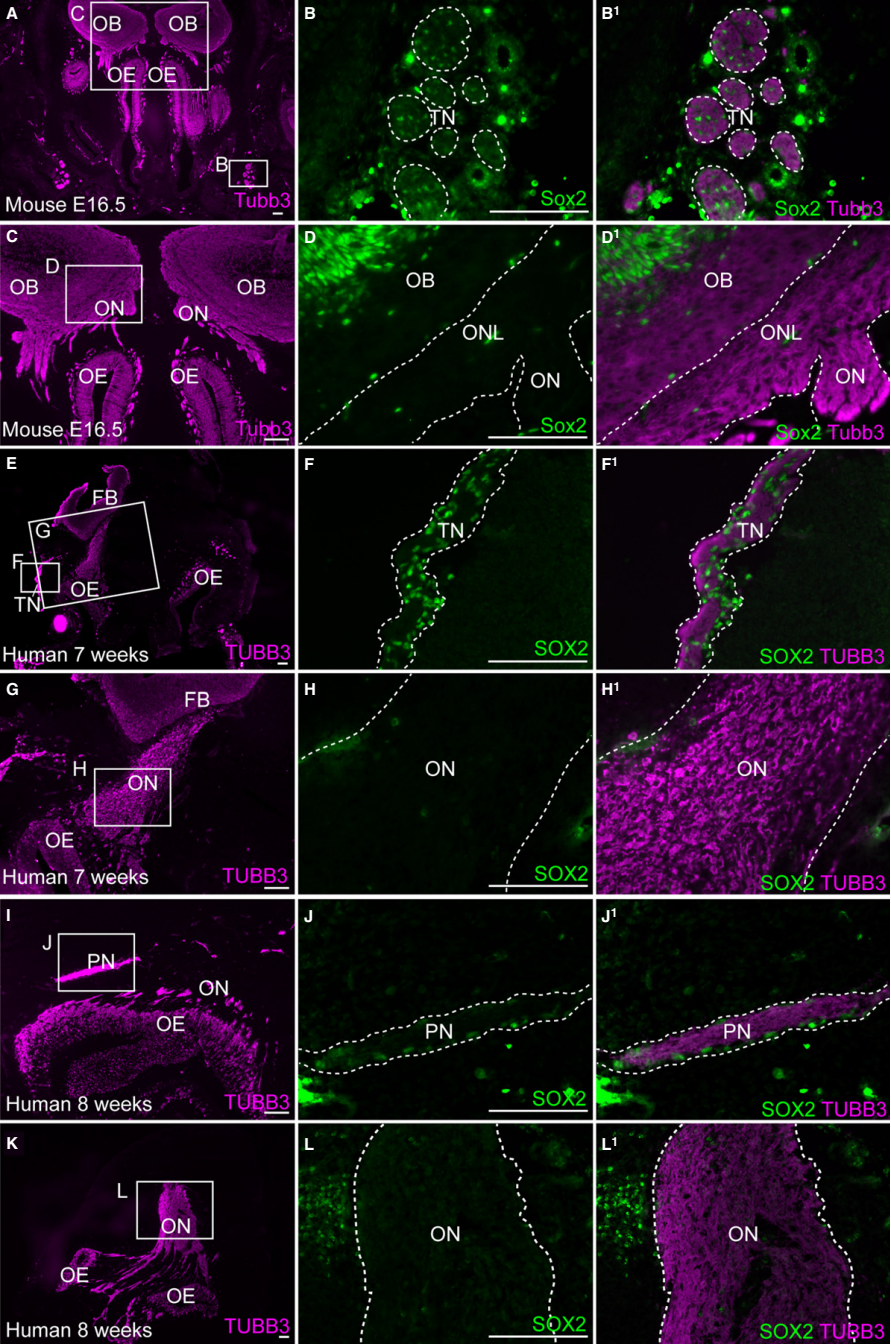
Embryonic human and mouse OECs do not express the Schwann cell precursor and immature Schwann cell marker Sox2. Coronal sections of the embryonic mouse (A–D^1^) and human (E–L) olfactory systems. (A) At E16.5 in the mouse, immunostaining for Tubb3‐positive axons provides an overview of the olfactory system. (B,B^1^) Higher‐power view of boxed region in (A), showing Sox2 expression in cells associated with Tubb3‐positive trigeminal axons, presumably developing Schwann cells. (C–D^1^) In contrast, on the same section shown in (A–B^1^), Sox2 is not expressed by OECs in the olfactory nerve layer of the olfactory bulb, or on the olfactory nerve. (E–F^1^) At 7 weeks of human development, SOX2 is strongly expressed by cells on the TUBB3‐positive trigeminal nerve, presumably developing Schwann cells, but (G–H^1^) SOX2 is not expressed by developing OECs on the olfactory nerve. (I–J^1^) Similarly at 8 weeks of human development, SOX2 is detected in developing Schwann cells, but (K–L^1^) is absent or expressed at very low levels in OECs on the same section. FB, forebrain; OB, olfactory bulb; OE, olfactory epithelium; ON, olfactory nerve; ONL, olfactory nerve layer; PN, peripheral nerve; TN, trigeminal nerve. Scale bar: 100 μm.

In E16.5 mouse embryos, Sox2 is expressed by immature Schwann cells on trigeminal nerves as expected (Fig. 7A–B^1^) but, in striking contrast to chicken embryos Sox2 expression could not be detected in OECs, whether on the olfactory nerve or in the olfactory nerve layer of the olfactory bulb (Fig. 7C–D^1^). Similarly, in human embryos at both 7 and 8 weeks of development, Sox2 is expressed by developing Schwann cells on peripheral nerves, but not by developing OECs on the olfactory nerve (Fig. 7E–L^1^).

## Discussion

The current investigation of Notch signalling pathway gene expression in developing OECs arose from its importance for Schwann cell development (Woodhoo et al. 2009). Activating Notch signalling in Schwann cell precursors (which can be distinguished from their neural crest precursors by expression of Mpz and Fabp7, amongst others; Woodhoo & Sommer, 2008; Jacob, 2015; Jessen et al. 2015) accelerated the formation of immature Schwann cells, while blocking Notch signalling in Schwann cell precursors delayed the formation of immature Schwann cells (Woodhoo et al. 2009). PCR of rat sciatic nerves showed that the receptor genes *Notch1–3* are all upregulated from E14 to E18, coinciding with the transition from Schwann cell precursors to immature Schwann cells (Woodhoo et al. 2009). Here, it was found that *Notch1* is similarly upregulated in developing chicken OECs starting with a few cells at E5.5, 2 days after the first Mpz‐positive cells can be identified on the developing olfactory nerve at E3.5 (Drapkin & Silverman, 1999). Canonical Notch activity is maintained in at least a subset of OECs until E10.5, as the cleaved Notch1 intracellular domain can be identified at E8.5, and expression of the Notch target genes *Hes4* and *Lfng* is also seen in subsets of developing OECs at E9.5 and E10.5, respectively. Also, *Notch1* expression was identified in mouse OECs at E14.5 and E16.5, and in human OECs at 7 and 8 weeks of development. In immature Schwann cells, Notch signalling actively inhibits myelination and thus must be downregulated for the terminal differentiation of myelinating Schwann cells (Woodhoo et al. 2009). PCR of non‐myelinating Schwann cells at postnatal day 5 in the mouse has identified the maintenance of Notch receptor and target gene expression, in contrast to myelinating Schwann cells from the same stage (Woodhoo et al. 2009). Since OECs are non‐myelinating in their normal environment, it is likely that they also maintain Notch signalling into postnatal development, and downregulation of this pathway is likely to be required for their ability to myelinate larger‐diameter axons both *in vitro* and *in vivo*, for example when transplanted into sites of CNS damage (reviewed by Roet & Verhaagen, 2014).

Upregulation of the Notch/Rbpj target gene *Sox2* (Wakamatsu et al. 2004; Ehm et al. 2010) was also identified in the chicken OEC lineage from E6.5. Sox2 is expressed during Schwann cell precursor and immature Schwann cell stages, but downregulation is necessary for Schwann cell maturation and myelination (Wakamatsu et al. 2004; Le et al. 2005; Adameyko et al. 2012; weak Sox2 expression is maintained in non‐myelinating Schwann cells; Koike et al. 2014). Sox2 is thought to play a key role in maintaining the Schwann cell precursor/immature Schwann cell state as cross‐regulatory suppression by the transcription factors Egr2 (Krox20) and Mitf results in Sox2 downregulation and commitment to, respectively, a myelinating Schwann cell (Egr2‐positive) fate or a melanocyte (Mitf‐positive) fate (Adameyko et al. 2012). The current results suggest that during OEC development in the chicken embryo, canonical Notch signalling mediates a transition from an early Mpz‐positive, Sox2‐negative ‘OEC precursor’ stage to a more mature Sox2‐positive stage. Sox2 is maintained in chicken OECs until at least E13.5: as this is several days after olfactory receptor neurons have begun to form synapses with their targets in the olfactory bulb, at E8–10 (Ayer‐Le Lièvre et al. 1995), OECs might be expected to be phenotypically mature by this stage. In the Schwann cell lineage, Sox2 already seems to have been downregulated by E13.5, presumably during the transition from immature to mature Schwann cells (Le et al. 2005). However, this seems not to be conserved in mammals, as Sox2 expression could not be detected in either mouse or human OECs, although it was present in developing Schwann cells.

Finally, it was found that olfactory receptor neurons in chicken, mouse and human embryos express the canonical Notch ligand gene *Jag1* at stages when *Notch1* is detected in developing OECs. (In chicken and mouse embryos, expression of *Jag2* was also identified in olfactory receptor neurons.) Similarly, Jag1 protein (detected by immunostaining) is expressed on sciatic nerve axons at E14 and at birth in the rat (although it is also present on Schwann cell precursors and Schwann cells) (Woodhoo et al. 2009). Thus, it is tempting to speculate that one or both of these ligands are available on olfactory axons to activate Notch1 in developing OECs and promote their maturation.

Overall, the current data support the existence of a transition in OEC development from an early Mpz‐positive ‘OEC precursor’ to a more mature stage, mediated by canonical Notch1 signalling and potentially activated by Jag1 from adjacent olfactory axons. A deeper molecular understanding of normal OEC development is important not only for our understanding of how neural crest cells generate different glial subtypes (reviewed by Jacob, 2015), but also because it may be possible in the future to expand and differentiate the neural crest stem cells that persist in skin and hair follicles (Toma et al. 2001; Wong et al. 2006; Hunt et al. 2009, 2010) into patient‐specific OECs for CNS repair.

## Author contributions

C.V.H.B. and S.R.M. designed the study. S.R.M. performed almost all of the experiments, analysed the data and prepared the figures. S.R.M. and C.V.H.B. wrote the paper. S.N.P. helped with mouse gene cloning and contributed some of the *in situ* hybridisation and immunostaining data. C.B. dissected and genotyped the mouse embryos. S.R.W.S. dissected the human foetal tissue.

## References

[joa12494-bib-0001] Adameyko I , Lallemend F , Aquino JB , et al. (2009) Schwann cell precursors from nerve innervation are a cellular origin of melanocytes in skin. Cell 139, 366–379. 19837037 10.1016/j.cell.2009.07.049

[joa12494-bib-0002] Adameyko I , Lallemend F , Furlan A , et al. (2012) Sox2 and Mitf cross‐regulatory interactions consolidate progenitor and melanocyte lineages in the cranial neural crest. Development 139, 397–410. 22186729 10.1242/dev.065581 PMC4067268

[joa12494-bib-0003] Andersson ER , Sandberg R , Lendahl U (2011) Notch signaling: simplicity in design, versatility in function. Development 138, 3593–3612. 21828089 10.1242/dev.063610

[joa12494-bib-0004] Ayer‐Le Lièvre C , Lapointe F , Leibovici M (1995) Avian olfactory neurogenesis. Biol Cell 84, 25–34. 10.1006/dbio.1996.0100 8608858

[joa12494-bib-0005] Barraud P , Seferiadis AA , Tyson LD , et al. (2010) Neural crest origin of olfactory ensheathing glia. Proc Natl Acad Sci USA 107, 21 040–21 045. 10.1073/pnas.1012248107 PMC3000254 21078992

[joa12494-bib-0006] Barraud P , St John JA , Stolt CC , et al. (2013) Olfactory ensheathing glia are required for embryonic olfactory axon targeting and the migration of gonadotropin‐releasing hormone neurons. Biol Open 2, 750–759. 23862023 10.1242/bio.20135249 PMC3711043

[joa12494-bib-0007] Beites CL , Kawauchi S , Crocker CE , et al. (2005) Identification and molecular regulation of neural stem cells in the olfactory epithelium. Exp Cell Res 306, 309–316. 15925585 10.1016/j.yexcr.2005.03.027

[joa12494-bib-0008] Blanchart A , Martín‐López E , De Carlos JA , et al. (2011) Peripheral contributions to olfactory bulb cell populations (migrations towards the olfactory bulb). Glia 59, 278–292. 21125652 10.1002/glia.21100

[joa12494-bib-0009] Bossy J (1980) Development of olfactory and related structures in staged human embryos. Anat Embryol (Berl) 161, 225–236. 7469043 10.1007/BF00305346

[joa12494-bib-0010] Britsch S , Goerich DE , Riethmacher D , et al. (2001) The transcription factor Sox10 is a key regulator of peripheral glial development. Genes Dev 15, 66–78. 11156606 10.1101/gad.186601 PMC312607

[joa12494-bib-0011] Cau E , Casarosa S , Guillemot F (2002) Mash1 and Ngn1 control distinct steps of determination and differentiation in the olfactory sensory neuron lineage. Development 129, 1871–1880. 11934853 10.1242/dev.129.8.1871

[joa12494-bib-0012] Dong Z , Sinanan A , Parkinson D , et al. (1999) Schwann cell development in embryonic mouse nerves. J Neurosci Res 56, 334–348. 10340742 10.1002/(SICI)1097-4547(19990515)56:4<334::AID-JNR2>3.0.CO;2-#

[joa12494-bib-0013] Drapkin PT , Silverman A‐J (1999) Development of the chick olfactory nerve. Dev Dyn 214, 349–360. 10213390 10.1002/(SICI)1097-0177(199904)214:4<349::AID-AJA7>3.0.CO;2-E

[joa12494-bib-0014] Dyachuk V , Furlan A , Shahidi MK , et al. (2014) Parasympathetic neurons originate from nerve‐associated peripheral glial progenitors. Science 345, 82–87. 24925909 10.1126/science.1253281

[joa12494-bib-0015] Ehm O , Goritz C , Covic M , et al. (2010) RBPJkappa‐dependent signaling is essential for long‐term maintenance of neural stem cells in the adult hippocampus. J Neurosci 30, 13 794–13 807. 20943920 10.1523/JNEUROSCI.1567-10.2010 PMC6633732

[joa12494-bib-0016] Ekberg JAK , Amaya D , Mackay‐Sim A , et al. (2012) The migration of olfactory ensheathing cells during development and regeneration. Neurosignals 20, 147–158. 22456085 10.1159/000330895

[joa12494-bib-0017] Espinosa‐Medina I , Outin E , Picard CA , et al. (2014) Parasympathetic ganglia derive from Schwann cell precursors. Science 345, 87–90. 24925912 10.1126/science.1253286

[joa12494-bib-0018] Henrique D , Adam J , Myat A , et al. (1995) Expression of a *Delta* homologue in prospective neurons in the chick. Nature 375, 787–790. 7596411 10.1038/375787a0

[joa12494-bib-0019] Hirata H , Yoshiura S , Ohtsuka T , et al. (2002) Oscillatory expression of the bHLH factor Hes1 regulated by a negative feedback loop. Science 298, 840–843. 12399594 10.1126/science.1074560

[joa12494-bib-0020] Hori K , Sen A , Artavanis‐Tsakonas S (2013) Notch signaling at a glance. J Cell Sci 126, 2135–2140. 23729744 10.1242/jcs.127308 PMC3672934

[joa12494-bib-0021] Hunt DP , Jahoda C , Chandran S (2009) Multipotent skin‐derived precursors: from biology to clinical translation. Curr Opin Biotechnol 20, 522–530. 19896826 10.1016/j.copbio.2009.10.004

[joa12494-bib-0022] Hunt DP , Sajic M , Phillips H , et al. (2010) Origins of gliogenic stem cell populations within adult skin and bone marrow. Stem Cells Dev 19, 1055–1065. 20102260 10.1089/scd.2009.0371 PMC3136724

[joa12494-bib-0023] Jacob C (2015) Transcriptional control of neural crest specification into peripheral glia. Glia 63, 1883–1896. 10.1002/glia.22816 25752517

[joa12494-bib-0024] Jacob C , Lötscher P , Engler S , et al. (2014) HDAC1 and HDAC2 control the specification of neural crest cells into peripheral glia. J Neurosci 34, 6112–6122. 24760871 10.1523/JNEUROSCI.5212-13.2014 PMC3996228

[joa12494-bib-0025] Jessen KR , Mirsky R (2005) The origin and development of glial cells in peripheral nerves. Nat Rev Neurosci 6, 671–682. 16136171 10.1038/nrn1746

[joa12494-bib-0026] Jessen KR , Brennan A , Morgan L , et al. (1994) The Schwann cell precursor and its fate: a study of cell death and differentiation during gliogenesis in rat embryonic nerves. Neuron 12, 509–527. 8155318 10.1016/0896-6273(94)90209-7

[joa12494-bib-0027] Jessen KR , Mirsky R , Lloyd AC (2015) Schwann cells: development and role in nerve repair. Cold Spring Harb Perspect Biol 7, a020487. 25957303 10.1101/cshperspect.a020487 PMC4484967

[joa12494-bib-0028] Joseph NM , Mukouyama YS , Mosher JT , et al. (2004) Neural crest stem cells undergo multilineage differentiation in developing peripheral nerves to generate endoneurial fibroblasts in addition to Schwann cells. Development 131, 5599–5612. 15496445 10.1242/dev.01429 PMC2638001

[joa12494-bib-0029] Kageyama R , Ohtsuka T , Kobayashi T (2007) The Hes gene family: repressors and oscillators that orchestrate embryogenesis. Development 134, 1243–1251. 17329370 10.1242/dev.000786

[joa12494-bib-0030] Kaukua N , Shahidi MK , Konstantinidou C , et al. (2014) Glial origin of mesenchymal stem cells in a tooth model system. Nature, 513, 551–554. 25079316 10.1038/nature13536

[joa12494-bib-0031] Kibbe WA (2007) OligoCalc: an online oligonucleotide properties calculator. Nucleic Acids Res 35, W43–W46. 17452344 10.1093/nar/gkm234 PMC1933198

[joa12494-bib-0032] Kobayashi T , Kageyama R (2014) Expression dynamics and functions of Hes factors in development and diseases. Curr Top Dev Biol 110, 263–283. 25248479 10.1016/B978-0-12-405943-6.00007-5

[joa12494-bib-0033] Koike T , Wakabayashi T , Mori T , et al. (2014) Sox2 in the adult rat sensory nervous system. Histochem Cell Biol 141, 301–309. 24170317 10.1007/s00418-013-1158-x

[joa12494-bib-0034] Kurtz A , Zimmer A , Schnutgen F , et al. (1994) The expression pattern of a novel gene encoding brain‐fatty acid binding protein correlates with neuronal and glial cell development. Development 120, 2637–2649. 7956838 10.1242/dev.120.9.2637

[joa12494-bib-0035] Lassiter RN , Dude CM , Reynolds SB , et al. (2007) Canonical Wnt signaling is required for ophthalmic trigeminal placode cell fate determination and maintenance. Dev Biol 308, 392–406. 17604017 10.1016/j.ydbio.2007.05.032 PMC3983986

[joa12494-bib-0036] Le N , Nagarajan R , Wang JYT , et al. (2005) Analysis of congenital hypomyelinating Egr2Lo/Lo nerves identifies Sox2 as an inhibitor of Schwann cell differentiation and myelination. Proc Natl Acad Sci USA 102, 2596–2601. 15695336 10.1073/pnas.0407836102 PMC548989

[joa12494-bib-0037] Lee M‐J , Calle E , Brennan A , et al. (2001) In early development of the rat mRNA for the major myelin protein P(0) is expressed in nonsensory areas of the embryonic inner ear, notochord, enteric nervous system, and olfactory ensheathing cells. Dev Dyn 222, 40–51. 11507768 10.1002/dvdy.1165

[joa12494-bib-0038] Li Y , Hibbs MA , Gard AL , et al. (2012) Genome‐wide analysis of N1ICD/RBPJ targets *in vivo* reveals direct transcriptional regulation of Wnt, SHH, and hippo pathway effectors by Notch1. Stem Cells 30, 741–752. 22232070 10.1002/stem.1030 PMC3734558

[joa12494-bib-0039] McKenzie IA , Biernaskie J , Toma JG , et al. (2006) Skin‐derived precursors generate myelinating Schwann cells for the injured and dysmyelinated nervous system. J Neurosci 26, 6651–6660. 16775154 10.1523/JNEUROSCI.1007-06.2006 PMC6674039

[joa12494-bib-0040] Murdoch B , Roskams AJ (2007) Olfactory epithelium progenitors: insights from transgenic mice and *in vitro* biology. J Mol Histol 38, 581–599. 17851769 10.1007/s10735-007-9141-2

[joa12494-bib-0041] Norgren RB Jr , Ratner N , Brackenbury R (1992) Development of olfactory nerve glia defined by a monoclonal antibody specific for Schwann cells. Dev Dyn 194, 231–238. 1281697 10.1002/aja.1001940308

[joa12494-bib-0042] O'Neill P , McCole RB , Baker CVH (2007) A molecular analysis of neurogenic placode and cranial sensory ganglion development in the shark, *Scyliorhinus canicula* . Dev Biol 304, 156–181. 17234174 10.1016/j.ydbio.2006.12.029 PMC4988491

[joa12494-bib-0043] Palmeirim I , Henrique D , Ish‐Horowicz D , et al. (1997) Avian *hairy* gene expression identifies a molecular clock linked to vertebrate segmentation and somitogenesis. Cell 91, 639–648. 9393857 10.1016/s0092-8674(00)80451-1

[joa12494-bib-0044] Paratore C , Goerich DE , Suter U , et al. (2001) Survival and glial fate acquisition of neural crest cells are regulated by an interplay between the transcription factor Sox10 and extrinsic combinatorial signaling. Development 128, 3949–3961. 11641219 10.1242/dev.128.20.3949

[joa12494-bib-0045] Peirano RI , Goerich DE , Riethmacher D , et al. (2000) Protein zero gene expression is regulated by the glial transcription factor Sox10. Mol Cell Biol 20, 3198–3209. 10757804 10.1128/mcb.20.9.3198-3209.2000 PMC85614

[joa12494-bib-0046] Pingault V , Bodereau V , Baral V , et al. (2013) Loss‐of‐function mutations in *SOX10* cause Kallmann syndrome with deafness. Am J Hum Genet 92, 707–724. 23643381 10.1016/j.ajhg.2013.03.024 PMC3644631

[joa12494-bib-0047] Radtke C , Kocsis JD (2014) Olfactory‐ensheathing cell transplantation for peripheral nerve repair: update on recent developments. Cells Tissues Organs 200, 48–58. 25765445 10.1159/000369006

[joa12494-bib-0048] Roet KCD , Verhaagen J (2014) Understanding the neural repair‐promoting properties of olfactory ensheathing cells. Exp Neurol 261C, 594–609. 24842489 10.1016/j.expneurol.2014.05.007

[joa12494-bib-0049] Sabado V , Barraud P , Baker CVH , et al. (2012) Specification of GnRH‐1 neurons by antagonistic FGF and retinoic acid signaling. Dev Biol 362, 254–262. 22200593 10.1016/j.ydbio.2011.12.016 PMC4561506

[joa12494-bib-0050] Sakaue M , Sieber‐Blum M (2015) Human epidermal neural crest stem cells as a source of Schwann cells. Development 142, 3188–3197. 26251357 10.1242/dev.123034 PMC4582175

[joa12494-bib-0051] Takeuchi H , Haltiwanger RS (2014) Significance of glycosylation in Notch signaling. Biochem Biophys Res Commun 453, 235–242. 24909690 10.1016/j.bbrc.2014.05.115 PMC4254162

[joa12494-bib-0052] Toma JG , Akhavan M , Fernandes KJ , et al. (2001) Isolation of multipotent adult stem cells from the dermis of mammalian skin. Nat Cell Biol 3, 778–784. 11533656 10.1038/ncb0901-778

[joa12494-bib-0053] Wakamatsu Y , Endo Y , Osumi N , et al. (2004) Multiple roles of SOX2, an HMG‐box transcription factor in avian neural crest development. Dev Dyn 229, 74–86. 14699579 10.1002/dvdy.10498

[joa12494-bib-0054] Wong CE , Paratore C , Dours‐Zimmermann MT , et al. (2006) Neural crest‐derived cells with stem cell features can be traced back to multiple lineages in the adult skin. J Cell Biol 175, 1005–1015. 17158956 10.1083/jcb.200606062 PMC2064709

[joa12494-bib-0055] Woodhoo A , Sommer L (2008) Development of the Schwann cell lineage: from the neural crest to the myelinated nerve. Glia 56, 1481–1490. 18803317 10.1002/glia.20723

[joa12494-bib-0056] Woodhoo A , Alonso MB , Droggiti A , et al. (2009) Notch controls embryonic Schwann cell differentiation, postnatal myelination and adult plasticity. Nat Neurosci 12, 839–847. 19525946 10.1038/nn.2323 PMC2782951

[joa12494-bib-0057] Wray S (2010) From nose to brain: development of gonadotrophin‐releasing hormone‐1 neurones. J Neuroendocrinol 22, 743–753. 20646175 10.1111/j.1365-2826.2010.02034.x PMC2919238

[joa12494-bib-0058] Ye J , Coulouris G , Zaretskaya I , et al. (2012) Primer‐BLAST: a tool to design target‐specific primers for polymerase chain reaction. BMC Bioinformatics 13, 134. 22708584 10.1186/1471-2105-13-134 PMC3412702

